# AI In Dental Education: Upskilled, Differently Skilled, or Never Skilled?

**DOI:** 10.1111/eje.70304

**Published:** 2026-09-11

**Authors:** Barry F. A. Quinn

**Affiliations:** ^1^ School of Dentistry, The University of Liverpool Liverpool UK

**Keywords:** artificial intelligence, assessment, clinical reasoning, competence, curriculum, dental education

## Abstract

**Background:**

Artificial intelligence (AI) is being integrated rapidly into dental education, yet frameworks to guide its use while safeguarding foundational competence remain underdeveloped. Recent work in medical education has highlighted the risk of AI‐induced ‘never‐skilling’, whereby learners fail to develop core reasoning because AI repeatedly performs the cognitive work required for competence development.

**Aim:**

To apply the concepts of ‘upskilled’, ‘differently skilled’ and ‘never skilled’ to European dental education and explore their implications for curriculum design, assessment and governance.

**Main Arguments:**

‘Never skilled’ refers not to poor AI literacy, but to progression through training without acquiring the foundational biomedical, behavioural and clinical knowledge, and independent reasoning expected of a safe clinician. In contrast, ‘upskilled’ individuals extend existing competencies through critical AI use, while ‘differently skilled’ individuals possess strong foundational competence and collaborate effectively with AI through contextual judgement, educational expertise and ethical reasoning. AI is not inherently detrimental to learning; its impact depends on when and how it is introduced. A staged approach that prioritises AI‐independent competence before guided AI integration and supervised collaboration is proposed.

**Conclusions:**

Dental schools should protect foundational competence through staged AI integration, explicit AI‐independent milestones, aligned assessment strategies, and separate governance frameworks for educational and clinical AI. The distinction between upskilled, differently skilled, and never skilled offers a practical framework for ensuring AI augments rather than replaces competence development.

## 1 Introduction

Artificial intelligence, including large language models, image‐analysis systems and clinical decision‐support tools, is increasingly visible across health professions education [1, 2, 3, 4]. In dentistry, AI is already being explored in radiographic interpretation, caries and lesion detection, risk prediction, virtual patients, haptic simulation, personalised learning pathways and assessment support [1, 2, 5, 6]. Reviews and commentaries suggest that these tools may widen access to cases, accelerate feedback and support more adaptive educational design [1, 2, 5, 6].

At the same time, concerns about academic integrity, bias, data protection, over‐reliance and opacity are becoming more prominent [2, 3, 7]. Guidance from institutions and professional bodies has started to emerge, but much of it remains generic and not sufficiently tailored to the specific cognitive, psychomotor and professional outcomes expected in dental programmes [2, 3, 7]. A recent Perspective in Nature Medicine has sharpened this debate by introducing the concept of AI‐induced ‘never‐skilling’, defined as failure to develop foundational clinical reasoning because AI substitutes for the cognitive effort required to build it [4].

This commentary uses that conceptual development to reconsider AI use in dental education. The central argument is that European dental schools should explicitly distinguish between those who are upskilled, those who are differently skilled, and students who risk becoming never skilled if foundational knowledge and independent reasoning are not secured before AI becomes a routine cognitive prosthesis [2, 3, 4, 7]. In doing so, the commentary also addresses two clarifications that have emerged in the subsequent literature: that AI literacy in dental education now runs in both directions between faculty and students, [8, 9] and that educational and clinical applications of AI should be treated as related but distinct educational objects [10, 11].

### 1.1 A Note on Terminology: Three Senses of AI Literacy

Because the term ‘AI literacy’ is used inconsistently in the literature, we distinguish three senses throughout this commentary. General AI literacy refers to a conceptual understanding of what AI systems are and how they behave. Dental AI literacy refers to domain‐specific understanding of AI applications in dentistry, their evidence base and their limits. AI critical appraisal refers to the applied capacity to evaluate a specific AI output against clinical evidence, professional guidelines and the clinician's own independent reasoning. These three competencies overlap but are not interchangeable, and they map onto different points in the framework developed below: general AI literacy is a prerequisite; dental AI literacy is where much of the current curricular gap sits; [8] and AI critical appraisal is the competency that most directly protects against never‐skilling and mis‐skilling.

## 2 Never‐Skilling, Deskilling and Mis‐Skilling

The *Nature Medicine* Perspective distinguishes three related but different risks [4]. Never‐skilling refers to failure to develop foundational competence during formative training because AI performs too much of the underlying reasoning [4]. Deskilling refers to the erosion of previously established competence in experienced clinicians through prolonged reliance on AI [4]. Mis‐skilling refers to the acquisition of flawed reasoning patterns through uncritical acceptance of incorrect or biased AI outputs [4].

This distinction is especially helpful for dental education because the principal concern for students is not loss of already established expertise, but progression without ever having formed it properly [4]. A student who repeatedly uses AI to generate diagnoses, treatment plans, explanations of pathophysiology or justifications for management may appear fluent in AI‐rich tasks while lacking the durable knowledge structures needed for OSCEs, licensing assessments and independent patient care [1, 2, 4, 5]. The risk is therefore educationally deeper than simple overuse of a tool; it is failure of schema formation [4].

The argument is theoretical but not speculative in the casual sense. The perspective grounds never‐skilling in desirable difficulties, deliberate practice, cognitive load theory and the expertise‐reversal effect, and supports the concern with early empirical signals from non‐clinical settings [4]. The paper also emphasises that AI is not harmful in itself; what matters is whether it is introduced as answer delivery during the stage when learners still need to build their own reasoning architecture, or used later in learning mode to stimulate reflection, calibration and critique [4].

## 3 Guarding Against Mis‐Skilling

Alongside never‐skilling, we must be careful not to mis‐skill our students. Mis‐skilling is the acquisition of incorrect clinical reasoning patterns through uncritical adoption of erroneous or biased AI outputs [4]. In this pattern, clinical schemas become contaminated by AI errors or bias, leading to systematic distortion of diagnostic or management reasoning [4]. In dental education, this is a particular concern wherever learners accept AI‐generated interpretations, differentials or treatment plans without independent verification. The risk is closely related to, but distinct from, the confidently generated but factually incorrect content produced by generative models (hallucination), and the impression of transparency created by explainability methods that have not been externally validated; both are considered explicitly below. The risk takes several recognisable forms: [4].

- Confidently incorrect differential diagnoses aligned with AI output.
- Internalisation of biased diagnostic associations (e.g., race or gender bias).
- Repeated reproduction of AI‐generated errors in independent reasoning.
- Occurs across all training stages when AI outputs are accepted without verification.

Because mis‐skilling can occur at any training stage, not only in early learners, it reinforces the need for verification, calibration and critical appraisal to be modelled and assessed throughout the curriculum rather than assumed [4].

## 4 Meaning of Never Skilled in Dental Education

In this commentary, ‘never skilled’ is used in a deliberately narrow and important sense. It does not mean a student who is poor at prompting, uninterested in digital tools or sceptical about AI. Rather, a never‐skilled student or graduate is one who has progressed through training without acquiring the foundational biomedical, behavioural and clinical knowledge, and without developing the independent reasoning capacity expected of a clinician, because AI has repeatedly performed those cognitive tasks on their behalf [4].

In dentistry, that risk may be especially visible in radiographic interpretation, caries‐risk assessment, diagnosis, treatment prioritisation, drafting of clinical notes, and formulation of prosthodontic or restorative treatment plans [1, 2, 5, 6]. If students habitually outsource these processes before attempting them independently, they may never consolidate the underlying concepts and patterns needed for safe practice [4]. The central educational failure is therefore not low general AI literacy, but progression without AI‐independent competence [4].

This interpretation matters because it shifts responsibility away from blaming individual learners and towards curriculum design, assessment architecture and institutional governance [4, 7]. If a programme allows students to advance while their apparent performance is contingent on AI availability, then the programme itself may be producing never‐skilled graduates [4].

### 4.1 The AI‐Literacy Inversion and Its Implications for Curriculum Design

A further complication, not fully captured in the original never‐skilling account, is that AI literacy in dental education can no longer be treated as a single deficit residing in the student. In many settings the gradient now runs in the opposite direction. A multinational survey of 508 dental students reported that 76.2% understood what AI entails and 67.4% were familiar with generic AI tools, yet only 34.7% understood AI's dental applications and 53.7% felt inadequately prepared by their curriculum [8]. A parallel study of students and dentists found that only 3% cited formal university education as their primary source of AI knowledge, whereas 64.7% relied on social media [9]. The educational task is therefore not introducing AI itself but developing dental AI literacy and AI critical appraisal, alongside responsible clinical application.

This changes what upskilling and differently skilled practice mean for faculty. Educators do not need to become the most technically fluent users of AI in the room, and in many cases they will not be. Their distinctive contribution is clinical reasoning, evidence interpretation, ethical and professional judgment and pedagogic design. That is precisely the differently skilled role. It also means the phased integration framework should not be read as faculty teaching students how to use AI. It is better read as a collaborative curriculum in which technical familiarity and disciplinary expertise are contributed by different members of the learning community and integrated through structured educational activity.

A concrete curricular consequence is that AI integration is not well served by adding a lecture series to an already crowded curriculum. A flipped‐classroom pattern is more coherent with the framework proposed here. Students undertake AI‐assisted preparatory activity outside class, generating draft differentials, treatment plans, patient explanations or interpretations of imaging. Classroom time is then devoted to faculty‐led critical appraisal of those outputs against clinical evidence, professional guidelines and the student's own reasoning. The educator's role shifts from teaching the mechanics of AI towards teaching clinical reasoning, evidence evaluation and professional judgement in an AI‐rich environment.

Faculty development is a prerequisite rather than an afterthought. Curricula designed around collaborative appraisal of AI outputs will fail if educators have not themselves been prepared to recognise hallucinations, biased associations and superficially plausible reasoning. Institutions therefore need parallel investment in faculty AI literacy—targeted specifically at critical appraisal of dental AI outputs rather than general AI awareness—before, or alongside, curricular change [8, 9].

## 5 Upskilled, Differently Skilled and Never Skilled

For strategic planning, it is useful to distinguish three overlapping groups within dental education [2, 3, 7].

- Upskilled: individuals who extend existing digital, clinical and pedagogic skills to incorporate AI critically into teaching, learning, assessment or administration [2, 5, 6].
- Differently skilled: individuals who possess sound foundational knowledge and independent reasoning, but whose strengths lie in using AI collaboratively, selectively and constructively through ethics, governance, pedagogy, communication, curriculum design or contextual judgement [4, 7].
- Never skilled: in student terms, learners who have not developed the foundational knowledge and independent reasoning required of a clinician because AI has repeatedly substituted for that learning work [4].

These are not value judgements. They are planning categories that help avoid the unrealistic assumption that every member of faculty and every student should become an advanced technical AI user [2, 3, 7]. More importantly, they redirect attention towards what must be protected: foundational competence for students, and diverse but valid forms of expertise among educators and professional services staff [2, 3, 4, 7].

## 6 Upskilled: The AI‐Literate Educator

The upskilled educator is the most familiar figure in current discussions of AI in higher education. Emerging dental and medical education literature suggests that such educators can use AI to support question writing, formative feedback, virtual patients, adaptive learning activities and interpretation of educational data [1, 5, 12]. In simulation and haptic environments, AI may also support objective feedback on manual performance and pattern recognition [1, 5].

An upskilled dental educator should not merely know how to operate tools. The more important capability is educational judgement: Deciding when AI supports learning and when it prematurely substitutes for it [4, 5]. This includes aligning AI with explicit outcomes, avoiding answer‐delivery mode in novice learners, and modelling how AI outputs should be verified against independent clinical reasoning and external evidence [1, 4, 5].

Upskilling therefore builds on established competence in digital radiography, CAD/CAM, electronic records, assessment design and simulation, but adds AI‐specific appraisal, calibration and ethical use [1, 2, 5].

## 7 Differently Skilled: Foundationally Competent, Collaborating With AI

The term differently skilled should not imply a lack of foundational knowledge. On the contrary, differently skilled educators and learners are already competent in the relevant biomedical, behavioural, clinical or educational domains; their distinctiveness lies in how they work with AI rather than whether they can use it at all [4, 7]. The Nature Medicine perspective is helpful here because it argues that the educational goal of human‐AI collaboration is not simple machine checking, but contribution of contextual reasoning, judgement and interpretation that AI alone cannot provide [4].

For educators, differently skilled practice might include using AI to generate alternative clinical vignettes, explore variations of item stems, map policy language, rehearse feedback phrasing or surface contrasting stakeholder perspectives, while relying on existing pedagogic, ethical or professional knowledge to critique and refine the output [2, 3, 7]. Educationalists and assessment leads may use AI to stress‐test blueprint coverage or marking criteria; governance leads may use it to compare draft policy statements; student support teams may use AI‐assisted analytics to identify patterns that still require human interpretation and local pastoral understanding [4, 7].

Differently skilled students also exist. These are learners with solid foundational knowledge and independent reasoning whose strengths lie in reflective practice, communication, contextual interpretation or critique rather than technical AI fluency. They may use AI constructively to rehearse explanations, compare alternative rationales, generate patient‐education drafts or interrogate treatment options, but their own knowledge remains primary [4]. The crucial distinction from never‐skilled students is that differently skilled learners can evaluate, contextualise and reshape AI outputs because they already possess the conceptual grounding to do so [4]. AI augments their work; it does not replace the formation of competence [4].

This distinction matters because it broadens the conversation beyond a narrow, tool‐centred definition of AI literacy. It recognises that constructive AI collaboration depends as much on prior disciplinary competence, ethical awareness and pedagogic judgement as on technical proficiency [2, 4, 7].

## 8 Hallucination, Explainability and the Learner Framework

Two related but distinct phenomena warrant more explicit discussion. Hallucination refers to confidently generated but incorrect content from language models, typically arising when a model produces plausible text without a factual basis. Explainable AI (XAI) refers to methods that make model reasoning inspectable, particularly in image‐based diagnostic tasks. The two are related because both concern trust, but they operate at different points in the workflow: hallucination is a property of the output, whereas explainability is a property of how the output can be examined.

Both connect directly to the learner framework. A never‐skilled learner, by definition lacking the foundational knowledge to evaluate a plausible answer, is disproportionately exposed to hallucinated content because they have no independent reference against which to test it. A differently skilled learner is exposed to a subtler risk: accepting a well‐explained but incorrect output because the explanation is coherent, especially where XAI outputs (e.g., saliency overlays on radiographs) create an impression of transparency that has not been externally validated. Making this connection explicit clarifies that verification and calibration are not general study skills but specific safeguards against these two failure modes.

The main families of XAI methods now appearing in dental research include image‐based saliency methods such as Grad‐CAM, feature‐attribution methods such as SHAP and local approximation methods such as LIME [10]. These are technically informative, but they should not be presented as a solution to the trust problem. A recent systematic review of dental XAI identified nineteen studies, of which fourteen had a high risk of bias, driven by small retrospective datasets, absent external validation and weak reference standards; only one study evaluated the impact of XAI on human understanding [10]. The authors conclude that dental XAI remains at a proof‐of‐concept stage and has not yet substantiated claims that it improves clinical trust or decision‐making [10]. Curricula that treat explainability as a complete answer to opacity are therefore ahead of the current evidence base. In educational terms, XAI outputs should be treated as an additional artefact to be critically appraised rather than as a warrant of correctness.

## 9 Educational AI and Clinical AI Are Not the Same Tool

A recurring source of confusion in current commentary is the conflation of AI used to support learning with AI used in patient care. The two applications share underlying technology, but their purposes, risks and required competencies differ.

When AI supports learning, the primary objective is knowledge acquisition, calibration and long‐term competence. Errors have educational consequences but do not directly harm patients. The competencies to be developed are critical thinking, verification against evidence, prompt and query design and self‐directed learning behaviours.

When AI enters patient care, the calculus changes. AI outputs may inform diagnosis or treatment, become part of the clinical record, and carry regulatory, ethical and medico‐legal weight; responsibility remains with the clinician. The competencies required are correspondingly different: understanding of model validation and generalisability, recognition of dataset and demographic bias, awareness of regulatory oversight (including software‐as‐a‐medical‐device frameworks) and the discipline of appropriate human supervision.

Evidence in dentistry supports keeping these frames separate. A systematic review with functional meta‐synthesis of AI‐assisted radiographic caries detection in dental education found some improvements in lesion detection, but educational benefits were inconsistent, evidence certainty was very low, and there was little evidence of durable learning gains [11]. In other words, an AI system that improves diagnostic performance at the point of use does not necessarily improve the underlying learning and may, under some conditions, displace it. This is precisely the never‐skilling risk expressed at the level of a single competency.

The practical consequence is that institutions should not operate a single AI policy. Educational AI policy should specify acceptable educational use, expected verification behaviour, and the assessment conditions under which AI is and is not permitted. Clinical AI policy should restrict implementation to validated systems, define supervisory expectations, and require that AI‐assisted clinical activity is auditable. Programmatic assessment should continue to include tasks that evaluate independent clinical reasoning without AI assistance, so that the AI‐independent milestones described below remain observable.

## 10 A Staged, Competency‐Protective Framework

The most useful practical contribution from the paper by Ke et al. (2026) is its three‐phase competency‐protective framework [4]. Although developed for medicine, it translates well to dentistry because the underlying concern is shared: learners must first acquire independent competence before they can collaborate safely and critically with AI [4]. In the sub‐sections that follow, each phase is now paired with an explicit assessment focus, in response to reviewer feedback that assessment ultimately determines whether learners become upskilled or never‐skilled.

### 10.1 Phase 1: Foundational AI‐Independent Competence

Early learners should develop core biomedical and clinical reasoning without relying on AI to generate answers [4]. In dentistry, this includes activities such as radiographic interpretation, diagnosis, caries‐risk assessment, treatment sequencing and justification of management decisions [1, 2, 5]. Assessments at this stage should require explanation of reasoning rather than AI‐mediated output [4]. AI may still be used as a study aid, but not to perform the reasoning work students are supposed to develop for themselves [4].

*Assessment focus*. Unaided written and viva assessment; structured explanation of reasoning in OSCE stations; radiographic and treatment‐planning tasks with AI disabled; and reflective accounts that require students to articulate diagnostic reasoning in their own words. The purpose is to establish observable AI‐independent milestones before Phase 2 permissions are granted.

### 10.2 Phase 2: Guided Integration and Calibration

Once baseline competence has been demonstrated, AI can be introduced in learning mode through structured tasks designed to build calibration rather than dependence [4]. This could include asking students to identify and correct errors in AI‐generated reports or treatment plans, comparing their unaided performance with AI‐supported performance, or using conversational AI in a Socratic way that stimulates rather than replaces reasoning [4, 12]. The educational objective here is to cultivate the capacity to know when AI adds value, when it misleads and when it should be overridden [4].

*Assessment focus*. Error‐spotting tasks on AI‐generated reports or treatment plans; paired unaided‐versus‐AI‐supported performance comparisons with a written calibration commentary; and OSCE stations in which the candidate must justify overriding or accepting an AI recommendation. Assessment at this stage evaluates AI critical appraisal explicitly, rather than assuming it as a by‐product of exposure.

### 10.3 Phase 3: Supervised Collaborative Practice

In later years and early postgraduate settings, AI can be integrated into supervised clinical work [4]. At this stage, the key question is what the human clinician contributes beyond AI output: patient values, contextual judgement, hands‐on findings, integration across problems and professional responsibility for decisions [4]. That logic aligns strongly with the differently skilled concept, because it emphasises constructive collaboration grounded in prior competence rather than passive dependence [4]. AI‐related tasks within entrustable professional activities should therefore be contingent on prior demonstration of AI‐independent competence and calibration [4].

*Assessment focus*. Workplace‐based assessment focused on the human contribution beyond the AI output (patient values, contextual judgement, integration across problems); entrustable professional activities that are contingent on documented Phase 1 and Phase 2 attainment; and portfolio evidence of AI‐assisted clinical episodes with reflective critique. Consistent with the earlier distinction between educational and clinical AI, at least some Phase 3 assessment must continue to test independent clinical reasoning without AI assistance.

## 11 Policy, Accreditation and Equity

The implications extend beyond curriculum design. Authors such as Ke et al. (2026) argue that if learners progress through training without demonstrable AI‐independent competence, this may affect patient safety, licensing integrity, workforce resilience and equity [4]. Those concerns are transferable to dentistry. A graduate who functions well only when AI is available may struggle in high‐stakes examinations, digitally constrained environments, emergency situations or contexts in which AI recommendations are wrong or unavailable [4].

This also raises questions for accreditation and quality assurance. Current competency frameworks in dentistry do not yet specify clearly how AI‐independent competence should be demonstrated, or which skills can be safely delegated and which must remain independently mastered [2, 3, 7]. This is especially persuasive in arguing that these questions should be addressed before harm is conclusively proven, because never‐skilling may be structurally difficult to detect and reverse once embedded at cohort scale [4].

At the institutional level, this argues for two distinct policies rather than one. An educational AI policy should specify acceptable educational use, expected verification behaviours, faculty development requirements, and the assessment conditions under which AI is and is not permitted. A clinical AI policy should restrict clinical implementation to validated systems, define supervisory expectations, and require that AI‐assisted clinical activity is auditable [10, 11]. Programmatic assessment should retain tasks that evaluate independent clinical reasoning without AI assistance, so that AI‐independent milestones remain observable across the programme.

Institutionally, the upskilled/differently skilled/never skilled lens can support more realistic staff development and role expectations [13]. Not every valued educator needs to be a technically advanced AI user, but every programme does need clarity on where foundational competence must be protected, where AI collaboration is educationally constructive, and where governance safeguards are needed [2, 3, 4, 7].

## 12 Conclusion

AI is likely to become a permanent feature of dental education [1, 2, 5, 6, 14]. The more important question is not whether it should be used, but how it should be introduced so that students do not emerge as a never‐skilled generation whose foundational knowledge and reasoning were never fully formed because AI repeatedly carried that load [4].

Using the distinctions between upskilled, differently skilled and never skilled allows a more precise and humane educational response. It recognises the value of collaborative AI use when grounded in prior competence, while making clear that foundational knowledge and independent reasoning must be protected deliberately through staged integration, assessment design and governance [2, 3, 4, 7]. The framework becomes still more useful when it is coupled with three further clarifications: that AI literacy in dental education is now bidirectional between faculty and students, so curriculum redesign and faculty development must proceed together; [8, 9] that hallucination and explainability are distinct risks that map onto the learner categories in specific ways, and that current XAI evidence in dentistry does not yet warrant treating explainability as a solution; [10] and that educational and clinical AI should be governed by separate policies, informed by evidence that diagnostic performance gains do not automatically translate into durable learning gains [11]. In that sense, the key issue is not whether AI creates dependency, but which dependencies represent acceptable augmentation and which represent a failure to develop competence in the first place [4].

## Funding

The author has nothing to report.

## Conflicts of Interest

The author declares no conflicts of interest.

## Data Availability

Data sharing not applicable to this article as no datasets were generated or analysed during the current study.
